# Calcium Sulfate and Platelet-Rich Plasma make a novel osteoinductive biomaterial for bone regeneration

**DOI:** 10.1186/1479-5876-5-13

**Published:** 2007-03-07

**Authors:** Giuseppe Intini, Sebastiano Andreana, Francesco E Intini, Robert J Buhite, Libuse A Bobek

**Affiliations:** 1 Department of Oral Biology, University at Buffalo – 109 Foster Hall, 3435 Main Street, Buffalo, NY, 14214, USA; 2 Department of Periodontics and Endodontics, University at Buffalo – 250 Squire Hall, 3435 Main Street, Buffalo, NY, 14214, USA; 3 Private Practice – Via Napoli 5/B, 70015 Noci (BA), Italy; 4 Department of Restorative Dentistry, University at Buffalo – 215 Squire Hall, 3435 Main Street, Buffalo, NY, 14214, USA; 5 Department of Oral Maxillo-Facial Surgery, University at Buffalo – 112 Squire Hall, 3435 Main Street, Buffalo, NY, 14214, USA

## Abstract

**Background:**

With the present study we introduce a novel and simple biomaterial able to induce regeneration of bone. We theorized that nourishing a bone defect with calcium and with a large amount of activated platelets may initiate a series of biological processes that culminate in bone regeneration. Thus, we engineered CS-Platelet, a biomaterial based on the combination of Calcium Sulfate and Platelet-Rich Plasma in which Calcium Sulfate also acts as an activator of the platelets, therefore avoiding the need to activate the platelets with an agonist.

**Methods:**

First, we tested CS-Platelet in heterotopic (muscle) and orthotopic (bone) bone regeneration bioassays. We then utilized CS-Platelet in a variety of dental and craniofacial clinical cases, where regeneration of bone was needed.

**Results:**

The heterotopic bioassay showed formation of bone within the muscular tissue at the site of the implantation of CS-Platelet. Results of a quantitative orthotopic bioassay based on the rat calvaria critical size defect showed that only CS-Platelet and recombinant human BMP2 were able to induce a significant regeneration of bone. A non-human primate orthotopic bioassay also showed that CS-Platelet is completely resorbable. In all human clinical cases where CS-Platelet was used, a complete bone repair was achieved.

**Conclusion:**

This study showed that CS-Platelet is a novel biomaterial able to induce formation of bone in heterotopic and orthotopic sites, in orthotopic critical size bone defects, and in various clinical situations. The discovery of CS-Platelet may represent a cost-effective breakthrough in bone regenerative therapy and an alternative or an adjuvant to the current treatments.

## Background

On January 13, 2000 *The Bone and Joint Decade *was formally launched at the World Health Organization headquarters in Geneva, Switzerland [1,2]. The Bone and Joint Decade has been endorsed by the United Nations, 54 governments, and more than 750 organizations, medical groups, and journals worldwide. The ultimate goal is to improve quality of life for people with musculoskeletal conditions and to advance understanding and treatment of these conditions through research, prevention, and education. As musculoskeletal conditions are a leading cause of disability in the United States, costing the American society an estimated $254 billion every year [2], on March 21, 2002 President George W. Bush also signed a proclamation designating the years 2002–2011 as the Bone and Joint Decade in the United States [3]. The proclamation called upon the medical community "to pursue research in this important area." The present study represents a novel endeavor towards the development of innovative approaches in bone regenerative therapy.

Since Marshall Urist described bone formation by autoinduction [4] and by purified bone morphogenetic proteins (BMPs) from bone [5], researchers have focused their investigations on techniques for delivering BMPs in order to induce bone regeneration. However, while it is true that the delivery of recombinant BMPs promotes bone formation in animal models, it is also true that these results do not translate consistently into human clinical trials [6-8]. Indeed, the biology of the regeneration of bone is complex and involves the coordinated expression of growth factors, inhibitory factors, adhesive proteins and specific transcription factors [9]. For these reasons researchers are now employing other approaches such as the combinatorial delivery of multiple BMPs via gene therapy [10]. Clearly, the therapy of bone regeneration is still at its early stages and novel contributions are needed in order to develop efficient treatments.

With the present work we introduce CS-Platelet, a novel and simple biomaterial able to induce regeneration of bone in a variety of clinical situations. Differing from the approaches seen to date, in our work we hypothesized that nourishing a bone defect with calcium, a major inorganic constituent of bone, and with a large amount of activated platelets, representing a component of wound healing, may initiate a series of biological processes that culminate in bone regeneration. Indeed, platelets appropriately activated by an agonist such as thrombin or collagen release numerous biologically active factors that are involved in wound healing and in processes that culminate in parenchymal cell proliferation and tissue regeneration [11-15] (Table 1, Panel A). Thus, the appropriate delivery of activated platelets may represent a true combinatorial delivery of multiple biological factors for tissue regeneration.

**Table 1:** Panel A: List of the biologically active factors released by activated platelets. **Panel B**: Protocol for the preparation of PRP in each bioassay. The amount of blood collected, the centrifugal force, and the final PRP volume are shown.

**Panel A**					
**Biologically active factors released by the activated platelets**^11–15^					

ADP, ATP, Serotonin, Platelet-derived Growth Factor (PDGF), Transforming Growth Factor-β1 (TGF-β1), Connective Tissue Activating Peptide III (CATP III), Insuline-like Growth Factor-1 (IGF-1), Epidermal Growth Factor (EGF), Vascular Endothelial Growth Factor (VEGF), basic Fibroblast Growth Factor (bFGF), Angiopoietin-2 (Ang-2), Thrombospondin, Factor V, Factor XI, Factor XIII, Platelet-derived endothelial cell growth factor (PDECGF), Osteocalcin, and adhesive proteins such as Fibrinogen, Fibronectin, von Willebrand Factor (vWF), and P-Selectin.					

**Panel B**					

**Bioassay**	**Collected Blood (final volume, including anticoagulant)**	**Collection site**	**First spinning (RCF)**	**Second spinning (RCF)**	**PRP volume**

**Heterotopic bioassay (Ferret)**	5 ml	Jugular vein	40 g for 20'	235 g for 10'	250 μl
**Orthotopic bioassay (Rat)**	3 ml	Jugular vein	380 g for 2'15"	380 g for 5'	75 μl
**Orthotopic bioassay (Non-human primate)**	30 ml	Cephalic vein	1130 g for 2'15"	1130 g for 5'	1.5 ml
**Clinical cases (*Homo sapiens*)**	20 ml	Cephalic vein Basilic vein Median cubital vein	1130 g for 2'15"	1130 g for 5'	1 ml

Therefore, in our laboratories we have engineered CS-Platelet, a combination of Calcium Sulfate (CS), a reservoir of resorbable calcium, and Platelet-Rich Plasma (PRP), a pool of concentrated platelets suspended in plasma. Previous studies have shown that within CS-Platelet, CS is able to activate the platelets present in the PRP without the need for an agonist [16], and to carry and release growth factors in a time-dependent manner [17]. Thus, CS-Platelet is a candidate biomaterial for bone regeneration because it is able to sustain over time the nourishment of the bone defects via the combinatorial delivery of calcium and platelets' multiple biological factors. The main focus of the present study was to systematically test CS-Platelet in heterotopic (muscle) and quantitative orthotopic (bone) bone regeneration bioassays. In addition, to evaluate its clinical features, CS-Platelet was tested in a qualitative non-human orthotopic bioassay and in 5 dental and craniofacial clinical cases, where regeneration of bone was necessary.

## Methods

### Preparation of Platelet-Rich Plasma

**Figure 1:**
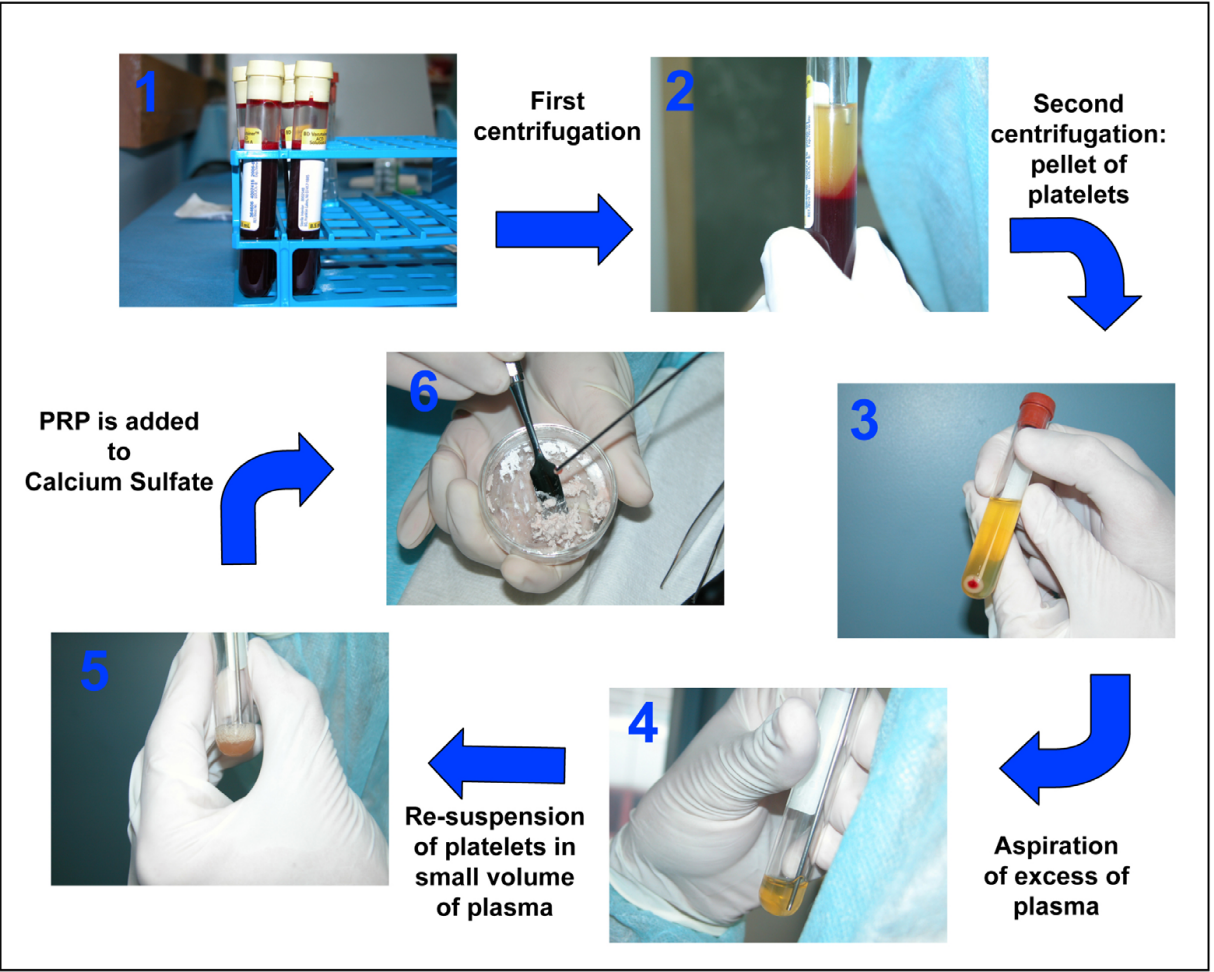
**Preparation of CS-Platelet: **Step 1: aliquots of whole blood (as indicated in Table 1) were collected in tubes containing acid-citrate-dextrose as an anti-coagulant. Step 2: immediately after being drawn, blood was centrifuged (see Table 1) to separate RBCs from platelets and plasma. Step 3: The supernatant composed of platelets and plasma was collected and centrifuged again (see Table 1) in order to pellet the platelets. Step 4 and 5: After this second centrifugation, the platelets were re-suspended in an appropriate volume of autologous plasma to achieve a platelet concentration 8–10 fold above the physiologic levels. (Step 6): CS (powder) was mixed with PRP (liquid) in a ratio of1 g of CS to 240 μl of PRP.

Platelet-Rich Plasma (PRP) is derived from autologous blood and is defined as a certain volume of plasma that has a platelet concentration several fold above the physiologic levels. In the present study we consistently used PRP with a high concentration of platelets (8–10 fold above the physiologic levels). The preparation of PRP, including the amount of blood collected, the centrifugal forces, and the final volume of the obtained PRP was optimized for each bioassay in order to obtain that concentration (see Table 1, Panel B for details). The concentration of platelets in whole blood and in the PRP was determined manually using a Petroff-Hausser counting chamber (improved Neubauer, cell-depth of 0.02 mm)(Hausser Scientific, USA). In all cases, PRP was prepared from autologous blood by a 2-step centrifugation process following previously published protocols [12,18] with some modifications (Fig. 1). Briefly, aliquots of whole blood were collected in tubes containing acid-citrate-dextrose as an anti-coagulant (0.163 ml per 1 ml of blood) (Fig. 1, step 1). Immediately after being drawn, blood was centrifuged to separate red blood cells (RBCs) from platelets and plasma (Fig. 1, step 2). Then, the supernatant composed of platelets and plasma was collected and centrifuged again in order to pellet the platelets (Fig. 1, step 3). After this second centrifugation, the platelets were re-suspended in an appropriate volume of plasma (Fig. 1, step 4, 5) to achieve a platelet concentration equal to 8–10 fold above the physiologic levels.

### Preparation of CS-Platelet

The commercially available powder form of Calcium Sulfate (CS) is the product of partial dehydration of gypsum, which produces CS hemihydrate (CaSO_4_·1/2 H_2_O). When the hemihydrate is mixed with water in the correct proportions, a suspension is formed that is initially fluid and workable; then, the hemihydrate completely precipitates until crystals of dihydrated solid CS are formed. This is an exothermic setting reaction during which 1 g of hemihydrate CS combines with 186 μl of water [19,20]. Similarly, as described in previous studies [16], CS-Platelet was prepared by mixing hemihydrate CS (Capset, Lifecore Biomedical, Inc. USA for the animal bioassays, and Surgiplaster, ClassImplant, s.r.l. Italy for the human clinical cases) with Platelet-Rich Plasma (PRP) in place of water. To account for the viscosity of plasma and for the platelet's volume, the powder (CS) to liquid (PRP) ratio was adjusted to 1 g of CS to 240 μl of PRP. By mixing CS with PRP a malleable paste was obtained and applied at the surgical sites (Fig. 1, step 6). After 30 minutes CS-Platelet crystallized becoming a solid and homogeneous compound [16]. According to each experiment or clinical case, the required amount of CS-Platelet was prepared by mixing CS with PRP in the ratio previously mentioned.

### Bioassays

All animal bioassays presented hereafter were conducted in accordance with the Institutional Animal Care and Use Committee (IACUC) of the University at Buffalo.

### Heterotopic (muscle) bioassay

**Figure 2:**
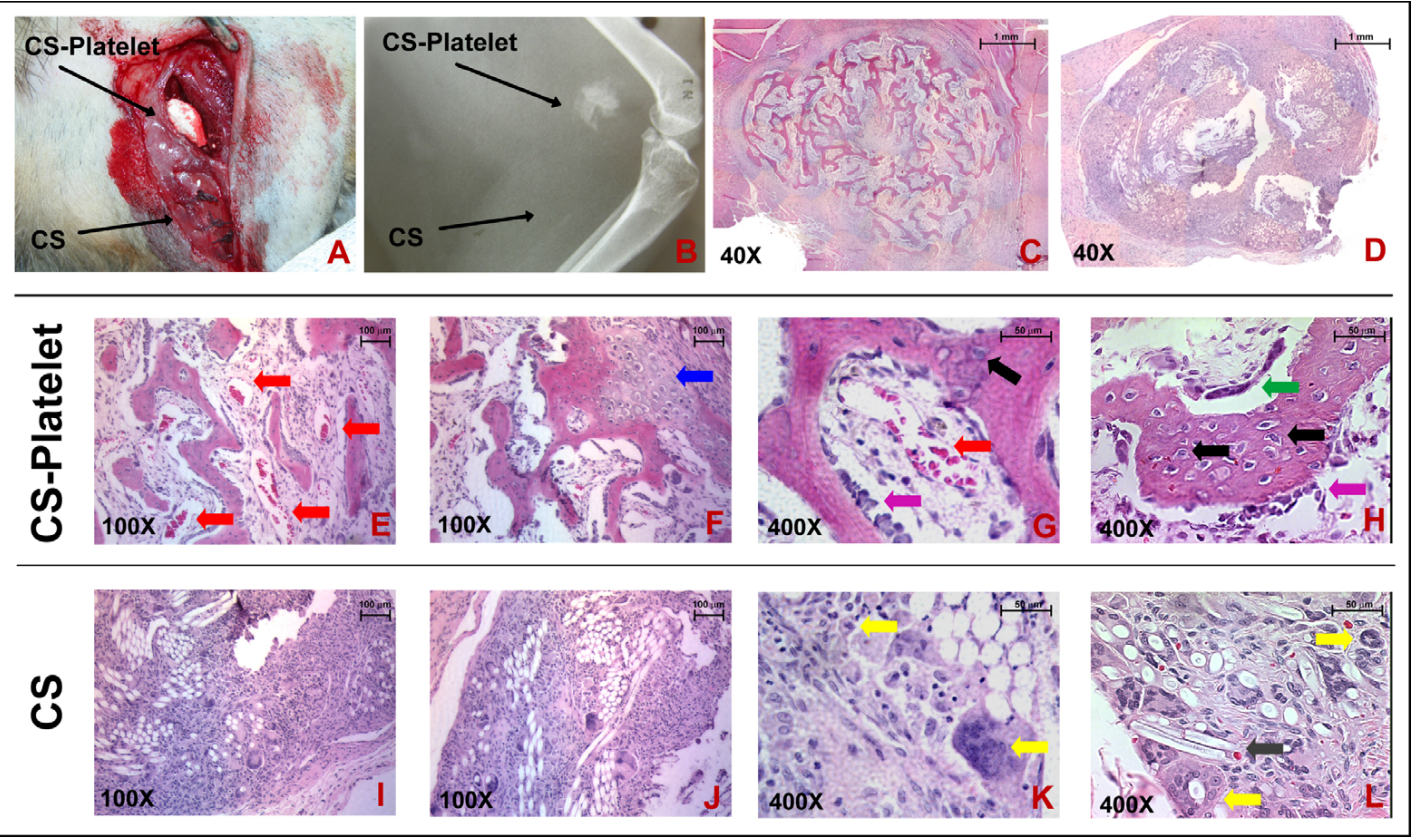
**Heterotopic (muscle) bioassay in ferrets: **(A): The biceps femoris of a ferret is shown. A sample of CS-Platelet and a sample of CS were implanted in the muscle tissue. (B): Four weeks after implantation the x-ray evaluations showed the presence of radio-opaque formations only in correlation with the implantations of CS-Platelet. (C, E-H): At the histological evaluation, the radio-opaque formation appeared as an ossicle formed by trabecular bone. (D, I-L): No bone formation was observed in correspondence with the implantations of Calcium Sulfate alone. Hematoxylin and Eosin staining.

A heterotopic bioassay [21,22] was used to test the osteoinductive activity of CS-Platelet (Fig. 2). To this end, 120 μl of PRP or 120 μl of double distilled sterile water were mixed with 500 mg of CS to prepare samples of CS-Platelet or CS. Then, 4 samples of CS-Platelet and 4 samples of CS were implanted into the triceps brachii and biceps femoris of a ferret (Fig. 2A). To help with post-surgical localization, a tattoo (1% India ink in 25 ul PBS) was performed at the mesial aspect of each implantation. Management of post-operative pain included subcutaneous administration of buprenorphine (0.01 mg/kg body weight). At 4 weeks post-implantation, x-rays were obtained from each muscle (10 MA, 78 kVp, 1/15 of a second impulse, anode-film distance = 20 cm) (Fig. 2B). Then, histological specimens were retrieved, demineralized in 14% EDTA for 10 days, and processed for tissue sectioning (4 μm) and staining (Hematoxylin and Eosin) (Fig. 2C–L).

### Rat orthotopic (bone) bioassay

**Figure 3:**
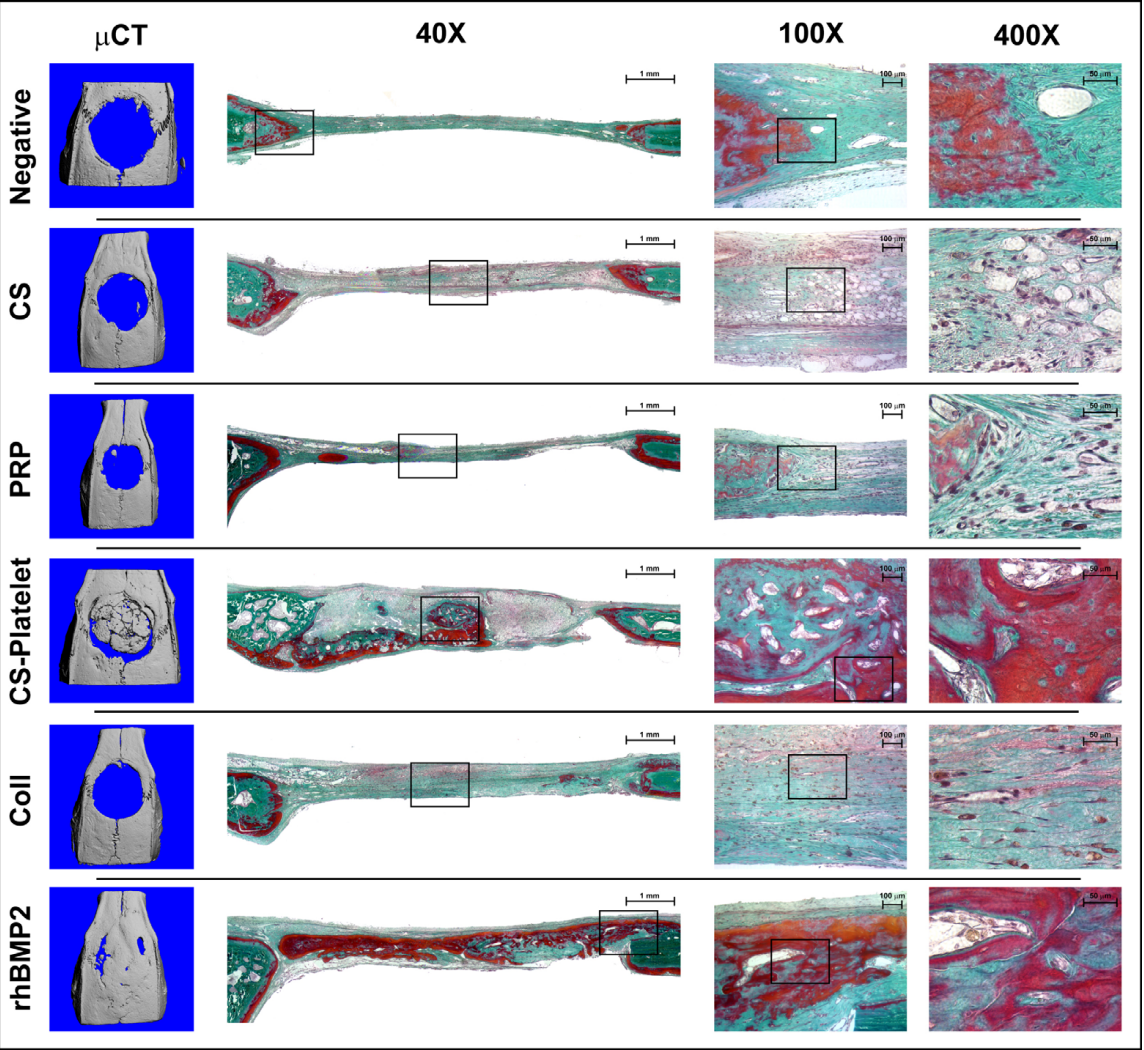
**Orthotopic (bone) bioassay in rats – From left to right: Microcomputed Tomography (μCT) images, sagittal histological sections, higher magnifications of histological sections (100× and 400× original magnifications)**. Eight weeks after implantation in the 8 mm rat critical size defects, only CS-Platelet and rhBMP2 showed a regeneration of bone extended at the center of the defects. In all other groups a limited regeneration of bone was seen only at the periphery of the defects. Goldner's trichrome staining: nuclear chromatin (brown-black), cytoplasm (bright red), erythrocytes (orange), collagen (light green), mineralized bone (green), osteoid (red).

**Figure 4:**
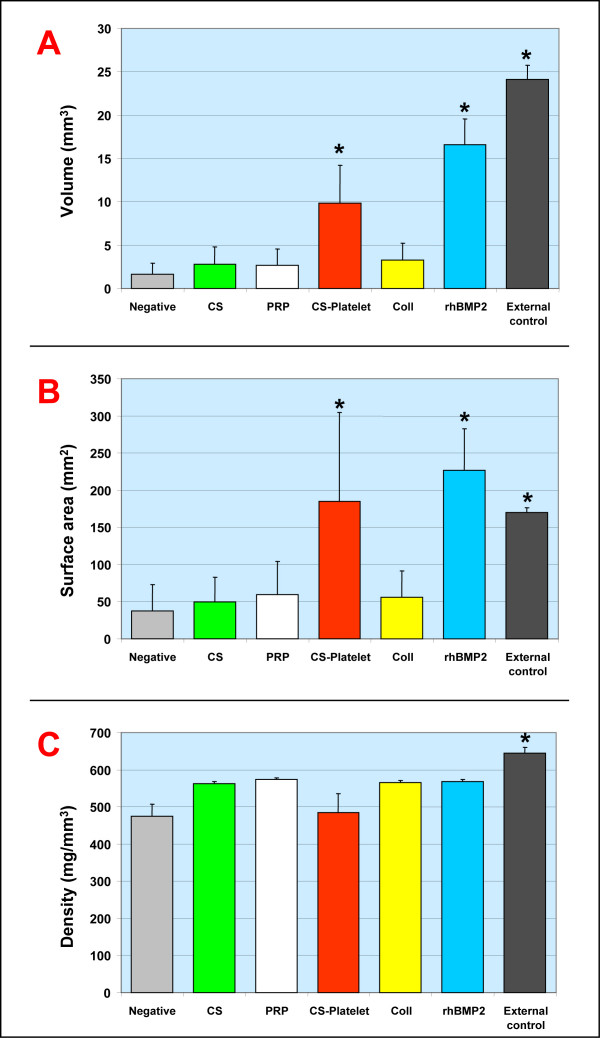
**Orthotopic (bone) bioassay in rats – Quantitative analysis of Tomography Data**. **Volume (A): **only CS-Platelet, rhBMP2, and the External control showed a statistically significant difference from the negative control (p < 0.001). **Surface area (B): **only CS-Platelet, rhBMP2, and the External control showed a statistically significant difference from the negative control (p < 0.001). **Density (C): **all treatment groups showed a density of the regenerated bone statistically different from the density of the bone normally present in an area of 8 mm of the calvaria (External control)(p < 0.001).

Using the 8 mm rat calvaria critical size defect (CSD) model of bone regeneration [23], the efficacy of CS-Platelet for *in vivo *bone regeneration was compared to the efficacy of other biomaterials (Fig. 3 and Fig. 4). The 8 mm diameter craniotomy defects were created with a trephine, avoiding perforation of the dura mater. Then, the created bone defects were filled with the chosen biomaterial. Briefly, 35 female adult Sprague-Dawley rats (275 g) were randomly divided into 7 groups of 5 rats each: 1) no filling biomaterial (Negative); 2) 100 mg of CS with 24 μl of double distilled sterile water (CS); 3) 24 μl of PRP activated by thrombin and CaCl_2 _in accordance with previously published protocols [12,24] (PRP); 4) 100 mg of CS and 24 μl of PRP (CS-Platelet); 5) 6.8 mg of collagen [25] (Coll); 6) 5 μg of human recombinant BMP2 (Cell Sciences, Inc. USA) and 6.8 mg of collagen [25,26] (rhBMP2); and 7) no surgery performed (External control). The rhBMP2 group (5 μg of rhBMP2 per cranial defect) represents a gold standard treatment for the regeneration of the 8 mm rat calvaria defect model [21,26] and in this study is used as comparative positive control. 100 mg of CS powder was determined to be the amount of CS required to completely fill the 8 mm cranial defect. After implantation of the biomaterials, the soft tissues were closed with skin staples. Management of post-operative pain included subcutaneous administration of buprenorphine (0.01 mg/kg body weight). Rats were euthanized by CO_2 _asphyxiation 8 weeks after surgery. The craniotomy sites with 10 mm contiguous bone were recovered from the skull and placed in a fixative solution (20 ml of RNA-Later, Ambion Inc, USA). All explanted calvaria were scanned using a Scanco Medical AG (Bassersdorf, Switzerland) μCT 40 system. Specimens were scanned using an 18 μm isotropic voxel size (FOV: 36.8; 2048 × 2048 image matrix) and 2000 projections/360 degrees [26]. Mineralized bone tissue was segmented from non-mineralized tissue by applying a gauss filter (sigma:1.6; support:3.0) and using a threshold of 21% of maximum gray scale value. The new bone formation was analyzed by extracting the region of new bone in the critical size defect using semi-automated contouring. The volume (mm^3^), the surface (mm^2^), and the density (mg/mm^3^) of the newly regenerated bone were measured by a blinded operator. Statistical power was calculated on previous bone regeneration calvaria defect studies [26] and differences among groups were statistically analyzed by ANOVA followed by the Fisher LSD multiple comparison test (significance level of 0.01). After tomography quantification, the specimens were processed for non-demineralized tissue sectioning (7 μm) and staining (Goldner's Trichrome).

### Non-human primate orthotopic (bone) bioassay

**Figure 5:**
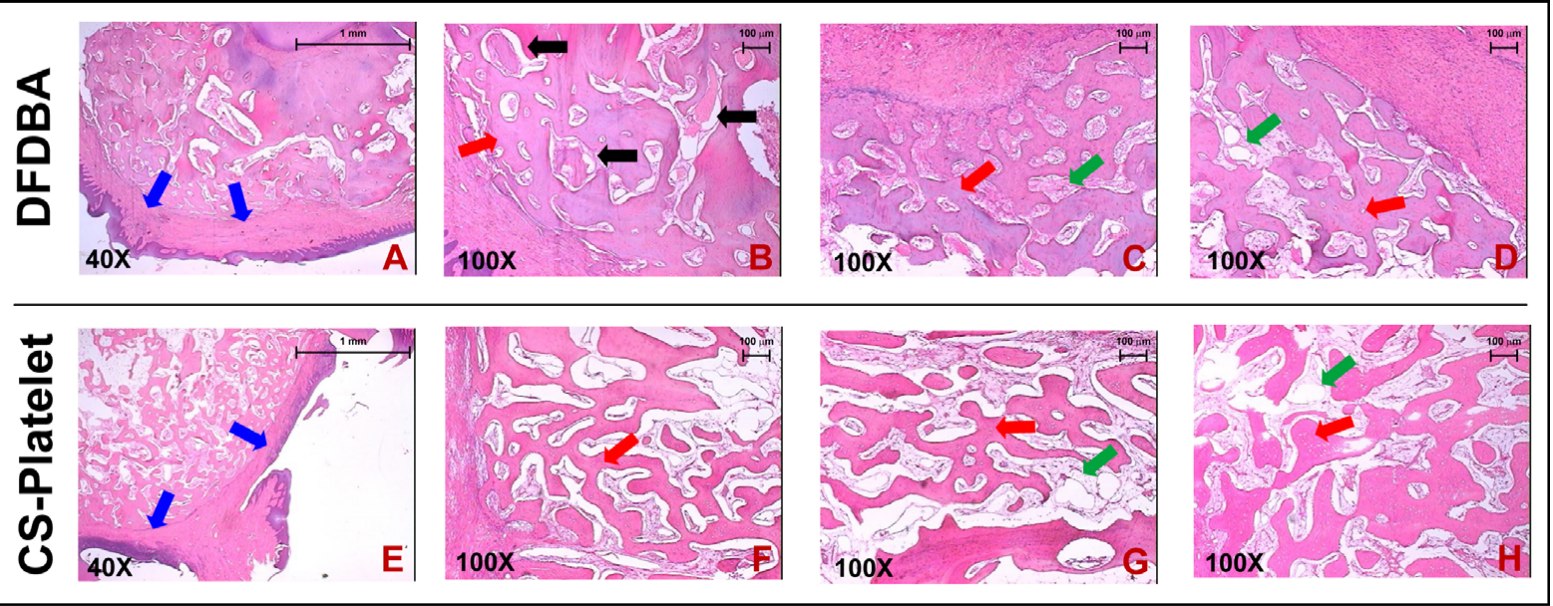
**Non-human primate orthotopic (bone) bioassay – Histology**. (A-D): samples of alveolar bone implanted with DFDBA. (E-H): Samples of alveolar bone implanted with CS-Platelet. All grafted sites, whether implanted with CS-Platelet or with DFDBA, showed regeneration of trabecular bone. In the sites grafted with DFDBA some residual biomaterial was still visible (B, black arrows). Hematoxylin and Eosin staining. (A and E, 40× original magnification). (B-D and F-H, 100× original magnification).

To test its clinical features and the histological outcomes, CS-Platelet was compared to demineralized freeze-dried bone allograft (DFDBA) (American Red Cross, Tissue Services, USA) (Fig. 5). One adult male non-human primate (*Macacus rhesus*) was used. Prior to the surgical extraction of the maxillary and mandibular first premolars, first molars, and third molars the animal was properly anesthetized. 30 ml of blood were collected for the preparation of 1.5 ml of PRP (see Table 1 for details). Then, using a split-mouth design, the right extraction sockets were each filled with CS-Platelet (1 g of Calcium Sulfate and 240 μl of PRP) and the left extraction sockets with human DFDBA (0.5 cc, American Red Cross – Tissue Services, Arlington VA). Management of post-operative pain included intramuscular administration of buprenorphine (0.01 mg/kg body weight) every 6–12 hrs for 4 days. Eight weeks after surgery, the animal was sacrificed and block sections of the grafted sites were collected, demineralized in 14% EDTA for 10 days, and processed for tissue sectioning (4 μm) and staining (Hematoxylin and Eosin).

### Human Clinical Cases

**Figure 6:**
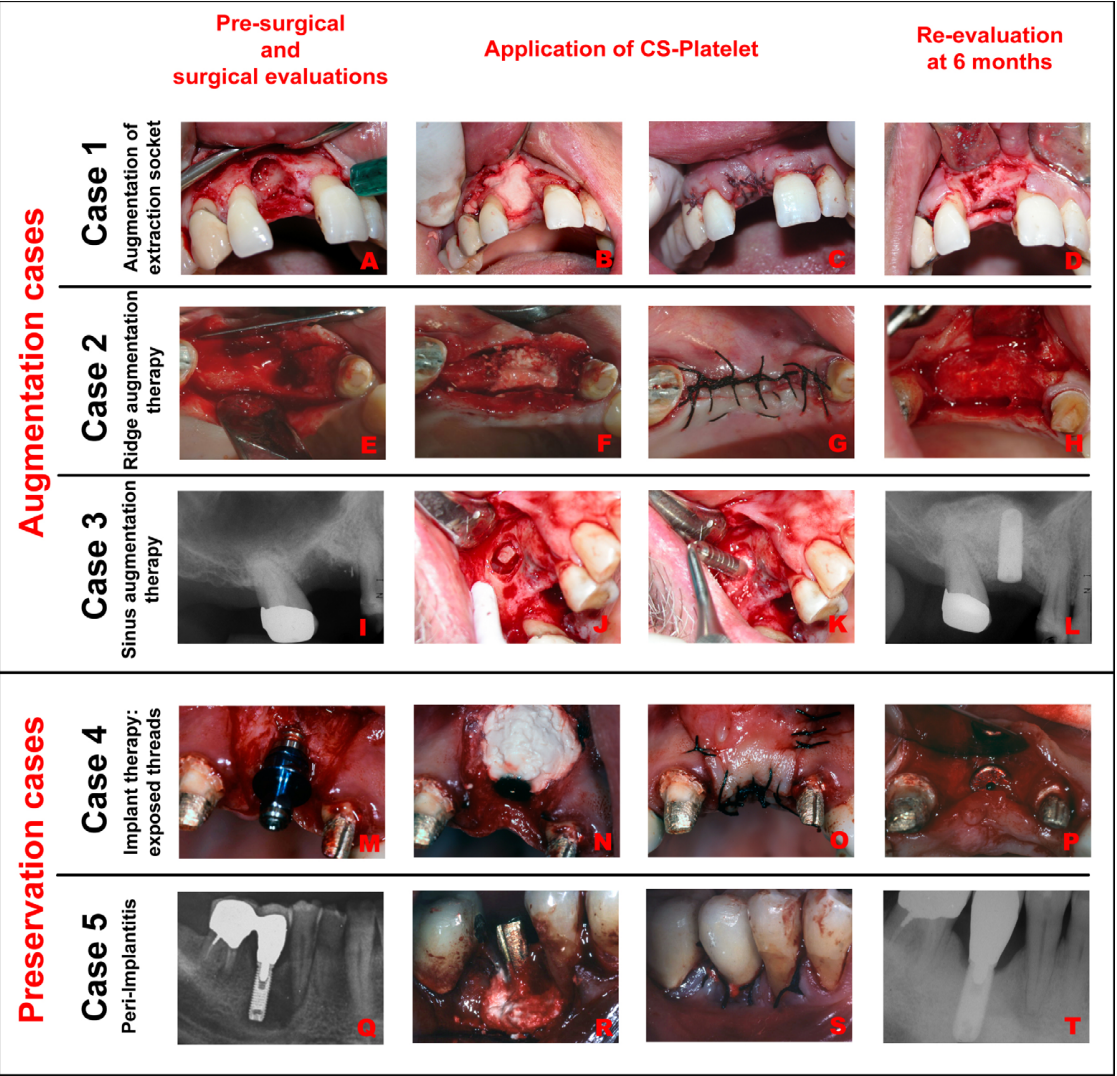
**Human clinical cases (from the top to the bottom). Augmentation cases: **(A-D): Augmentation of extraction socket upon extraction of tooth # 8. (E-H): Ridge augmentation therapy in area of teeth # 5–6. (I-L): Sinus augmentation therapy (induced bone formation within the right maxillary sinus) prior insertion of a titanium implant in area of teeth # 3–4. **Preservation cases: **(M-P): Regeneration of bone around the exposed threads of a titanium implant positioned in area of tooth # 9. (Q-T): Regeneration of bone around a titanium implant in position of tooth # 30 affected by peri-implantitis.

To test its clinical potentials, CS-Platelet was used in five human clinical dental cases (Fig. 6). In all patients treated (age group: 35–55 year old), the platelet count was within the normal limits and the medical and dental history revealed no significant contraindications to bone regenerative therapy. In all cases presented regeneration of bone was needed in order to either augment the amount of the preexisting bone for subsequent implantation of titanium implants (Augmentation cases: cases 1–3) or to augment the amount of bone surrounding a preexisting titanium implant to preserve the implant (Preservation cases: cases 4 and 5). Clinical cases consisted of: 1) Augmentation of extraction socket upon extraction of tooth # 8 (Fig. 6A–D), 2) Ridge augmentation therapy in area of teeth # 5–6 (Fig. 6E–H), 3) Sinus augmentation therapy (induced bone formation within the right maxillary sinus) prior insertion of a titanium implant in area of teeth # 3–4 (Fig. 6I–L), 4) Regeneration of bone around the exposed threads of a titanium implant positioned are of tooth # 9 (Fig. 6M–P), and 5) Peri-implantitis around titanium implant in position of tooth # 30 (Fig. 6Q–T). Prior to implantation of CS-Platelet the surgical recipient sites were freed of soft tissue tags and contaminated bone (tissue debridement). In the case of the sinus augmentation therapy, the schneiderian membrane was carefully elevated and CS-Platelet was implanted between the membrane and the floor of the sinus. In all other cases, after tissue debridement, CS-Platelet was simply applied in order to completely fill the bone defect. Then, the surgical wounds were sutured in order to achieve healing by first intention. Management of post-operative pain included administration of ibuprofen (400 mg t.i.d. p.r.n.). Clinical and radiographical re-evaluations were performed at 6 months. The human clinical cases were conducted in private dental practices on volunteers who gave written informed consent. Each subject was also informed that data concerning the case would be submitted for publication.

## Results

### Heterotopic (muscle) bioassay

Four weeks after implantation of CS or CS-Platelet in the triceps brachii and biceps femoris of a ferret (Fig. 2A), the x-ray evaluations showed the presence of radio-opaque formations only at the sites of the CS-Platelet implantations (Fig. 2B). Upon histological evaluation, bone formation in the form of an ossicle was noted (Fig. 2C and 2E–H). Highly vascularized trabecular bone associated with highly vascularized bone marrow rich of reticular cells was observed (Fig. 2E and 2G, red arrows). In addition, at the periphery of the bone nodule endochondral bone formation was noticeable (Fig. 2F, blue arrow), testifying a peripheral functional remodeling of mesenchymal tissue. No encapsulation of the bone nodule by chronic inflammation was observed. The presence of numerous active osteoblasts forming a rim of cells in the process of laying down osteoid (Fig. 2G and 2H, purple arrows), numerous osteocytes incorporated in the newly formed bone (Fig. 2G and 2H, black arrows), and osteoclasts forming Howship's lacunae (Fig. 2H, green arrow) confirm the high vitality of the generated bone nodule. No bone formation was observed at the site of the implantations of CS alone (Fig. 2D). CS was not completely resorbed and residual small crystals of CS were seen within the tissue. (Fig. 2L, grey arrow). The amount of residual CS is modest and the crystals are too dispersed to be shown by our x-ray evaluation (Fig. 2B). A foreign body reaction with both chronic (multinucleated-giant cells) and acute inflammatory response (polymorphonucleated) were also noticed (Fig. 2K,L, yellow arrows). Importantly, in both the CS-Platelet and CS cases, no signs of malignancy were observed.

### Rat orthotopic (bone) bioassay

Eight weeks after implantation in the 8 mm rat critical size defects, only CS-Platelet and rhBMP2 showed a regeneration of bone extended at the center of the defects (Fig. 3). In both these groups, areas of vascularized bone marrow are evident, and numerous osteocytes are present within the newly formed bone, testifying the vitality of the regenerated tissue. Osteoblasts present at the osteoid interface (osteoid tissue is stained in red with Goldner Trichrome) are in the process of laying down osteoid matrix. In all other groups, a limited regeneration of bone was seen only at the periphery of the defects (Fig. 3). As expected, in these cases bone regenerated only at the margins with fibrotic tissue always present at the center of the defects. In both the CS-Platelet and CS groups the implanted biomaterial is not completely resorbed (Fig. 3). Upon quantification and statistical analysis of the volume of bone regenerated within the 8 mm defect (Fig. 4A), only CS-Platelet, rhBMP2, and the External control showed a statistically significant difference from the negative control (p < 0.001). Evidently, CS alone (CS), activated PRP alone (PRP), and Collagen alone (Coll) are able to regenerate the same amount of bone that is regenerated when the defect is left unfilled (Negative)(non statistical significant difference). More specifically, CS-Platelet regenerated a volume of bone approximately 2/3 the volume regenerated by rhBMP2 (statistically significant difference, p < 0.001) and approximately 1/2 the volume present in an area of 8 mm of the normal calvaria (External control) (statistically significant difference, p < 0.001). Upon quantification and statistical analysis of the surface area of the bone regenerated within the 8 mm defect (Fig. 4B), only CS-Platelet, rhBMP2, and the External control showed a statistically significant difference from the negative control (p < 0.001). However, the surface area of the bone regenerated by CS-Platelet was not statistically different from the one regenerated by rhBMP2 (p = 0.26) and the one normally present in an area of 8 mm of the normal calvaria (External control) (p = 0.68). Again, CS alone (CS), activated PRP alone (PRP), and Collagen alone (Coll) are able to regenerate the same surface area of bone that is regenerated when the defect is left unfilled (Negative)(non statistically significant difference). Upon quantification and statistical analysis of the density of the bone regenerated within the 8 mm defect (Fig. 4C) all treatment groups showed a density of the regenerated bone statistically different from the density of the bone normally present in an area of 8 mm of the normal calvaria (External control)(p < 0.001).

### Non-human primate orthotopic (bone) bioassay

Eight weeks after implantation of CS-Platelet or demineralized freeze-dried bone allograft (DFDBA) in the tooth-extraction sockets, healing was shown to be uneventful and no bone loss or recession was reported at the adjacent teeth. In both cases, histologically, the overlying mucosa was fully formed with stratified squamous epithelium (Fig. 5A and 5E, blue arrows). Moreover, all grafted sites, whether implanted with CS-Platelet or with DFDBA, showed regeneration of trabecular bone (Fig. 5B–D and 5F–H, red arrows) associated with fibrofatty vascularized bone marrow (Fig. 5C–D and 5G–H, green arrows). Importantly, the grafted sites with DFDBA showed some residual biomaterial (Fig. 5A, black arrows), whereas no residual biomaterial was found in the sites grafted with CS-Platelet. In both cases, no signs of malignancy and no signs of chronic or acute inflammation were evident.

### Human Clinical Cases

Six months after implantation of CS-Platelet, the patients were seen for re-evaluation of the bone regenerative therapy (Fig. 6). In all augmentation cases (Cases 1–3), the surgical re-opening showed complete bone repair within the treated area so that placement of titanium implant was achievable and successful (Fig. 6D,H and 6L respectively). The radiographical exam, when present (Fig. 6L), also confirmed the clinical evaluation, showing an area of radio-opaque bone repair within the bone defect. The Exposed threads therapy and the Peri-Implantitis (Preservation cases: cases 4 and 5) showed a successful bone repair with the achievement of healthy clinical status that allowed for the preservation of the previously implanted titanium implants (Fig. 6P and 6T respectively).

## Discussion

Prior studies showed that CS and PRP, used separately, have limited degrees of success in bone regenerative therapy [27-33]. Although safely used in orthopedics for more than 100 years [27], CS alone has only been effective in treatment of non-critical (union) size bone defects [28,29]. More recently, CS has been proposed as a graft barrier rather than a true bone regenerative biomaterial [30,31,34]. PRP has shown some degree of effectiveness only when used in combination with certain biomaterials. For instance, Marx et al [24] showed that a significantly greater percentage of bone regeneration can be achieved when autogenous cancellous bone grafts were combined with PRP. Confirming the results of this study, another animal study showed that PRP may be beneficial when applied in combination with autologous bone [35]. A randomized, split mouth, double-masked clinical study [36] showed that the addition of PRP to a bovine derived xenograft to treat human intrabony defects significantly improved the clinical outcome. Another recent study evaluated the effect of PRP in the treatment of periodontal intrabony defects in humans [37] and showed that the combination of PRP with bovine porous bone mineral led to significantly favorable clinical improvements. The unfavorable results seen in other studies [32,33] may be due to the incompatible combination of PRP with the chosen biomaterial or to the fact that the concentration of platelets in PRP may vary according to the procedure used for its preparation [32]. It is still not clear whether low concentrations of platelets (2–3 fold above the physiologic levels) are more effective in regenerating bone than moderate (5 fold) or high (8–10 fold) concentrations, as recent *in vitro *studies [38,39] are inconsistent with previous *in vivo *studies [40]. In the present study we consistently used PRP with a concentration of platelets 8–10 fold above the physiologic levels.

Our work provides clear evidence that CS and PRP when opportunely combined (CS-Platelet) are able to induce regeneration of bone in critical size (nonunion) bone defects. This is due to the unique characteristics of CS-Platelet that CS alone and PRP alone do not possess. In fact, previous studies [16] showed that the exothermic reaction generated by the mixture of CS with PRP (CS-Platelet) activates the platelets contained within the PRP without the need for an agonist. Due to this activation process, the multiple biological factors released by the activated platelets are present in high concentrations and in their physiologically high ranges within CS-Platelet. The same studies also showed that CS-Platelet maintains all the optimal clinical advantages associated with CS, in that CS-Platelet is initially moldable and later solidifies, maintaining the space/scaffold needed by the bone regenerative process. Later [17] it was also proven that CS can slowly release recombinant human Platelet-Derived Growth Factor (rhPDGF-BB) over time, therefore supporting the hypothesis that CS-Platelet behaves as time-controlled releasing matrix for all the platelet derived factors. Because a major concern in delivery of the growth factors to the site of bone healing has always been their short half-lives [41], CS-Platelet represents a novel biocompatible delivery system for growth factors. Thus, CS-Platelet is a biomaterial that contains several components and qualities ideal for bone regeneration: 1) high quantities of calcium, 2) physiologically high concentrations of platelet's growth factors, 3) physiologically high concentrations of platelets' and plasma's adhesive proteins, 4) time-controlled release of growth factors, 5) initial malleability, 6) subsequent solidification, and 7) space maintaining properties. It is therefore not surprising that the heterotopic bioassay showed that CS-Platelet is an osteoinductive biomaterial (able to induce the formation of bone in muscle) as opposed to a simple osteoconductive biomaterial (serve only as scaffold for bone formation). In addition, the orthotopic bioassay in rats confirmed the osteoinductive quality of CS-Platelet. In nonunion defects CS-Platelet induced the formation of bone, in terms of volume, equal to approximately 2/3 the bone induced by the gold standard treatment (rhBMP2). Moreover, in terms of surface area of the regenerated bone, CS-Platelet and rhBMP2 are comparable and also equivalent to the surface area of the normal calvaria (External control). Yet, the density of the regenerated bone in both CS-Platelet and rhBMP2 groups is lower than that observed in normal calvaria (External control). This indicates that after 8 weeks of healing the regenerated bone is still substantially different from the normal bone and that a longer healing time may be needed to achieve full density. In our quantitative rat orthotopic bioassay, the volume of bone regenerated by the delivery of rhBMP2 exceeds the volume regenerated by CS-Platelet. This may be due to the fact that human rBMP2 when implanted in heterologous bone defects, such as those used in animal studies, is more effective [42]. Noticeably, it has also been reported that the successful use of human rBMPs in animals does not translate consistently into human clinical trials [6-8].

The quantitative orthotopic assay also showed that CS alone and the activated PRP alone are able to sustain only a minimal, peripheral, regeneration of bone. It is not surprising that CS alone and PRP alone are not able to induce any significant regeneration of bone when compared to a negative control. Clearly, CS lacks the growth factors provided by the PRP while PRP requires CS as an efficient carrier for its growth factors. The presence of a layer of bone on the bottom of the defect and the presence of a layer of un-resorbed CS-Platelet over the regenerated bone also shows that CS-Platelet acts as a functional barrier. A functional barrier, by impeding the migration of the epithelial cells from the skin into the bone defect, facilitates the bone regeneration process that is sustained only by osteocompetent cells [43]. Thus, we speculate that CS-Platelet, by acting as an epithelial barrier, facilitates the formation of bone that is sustained by the pervascular mesenchymal-type cells of the dura mater, on the bottom of the defect [44]. Importantly, although we did not measure the level of vascularization, in both the heterotopic and the orthotopic bioassays the qualitative histological evaluation of the CS-Platelet grafted sites consistently showed an elevated number of blood vessels. The ability of a biomaterial to induce vascularization is highly desirable in bone tissue engineering because it represents the means for nourishing and supporting the migration of competent cells.

The important conclusions of the present study are based on the heterotopic bioassay and on the quantitative rat orthotopic bioassay. However, in order to test the clinical features of CS-Platelet, we designed the qualitative orthotopic non-human bioassay and we tested CS-Platelet in some clinical cases. Significantly, we report that CS-Platelet in 6 tooth-extraction sockets of a non-human primate is completely resorbed after 8 weeks. This, together with no histological signs of malignancy or chronic and acute inflammation makes CS-Platelet a safe biomaterial suitable for various bone regenerative therapies. Confirming these conclusions, in the human clinical cases where CS-Platelet was tested we observed repair of hard tissues. The augmentation therapies showed reconstitution of the anatomy of the alveolar ridges and the preservation therapies showed the ability of CS-Platelet to treat bone loss around titanium implants. The clinical efficacy of CS-Platelet correlated well with its clinical viability as CS-Platelet, thanks to its physical attributes, was easy to prepare and simple to be applied within the bone defects.

## Conclusion

The heterotopic bioassay and the quantitative rat orthotopic bioassay allowed us to conclude that CS-Platelet is osteoinductive and regenerates bone in critical size bone defects. The orthotopic non-human bioassay and the clinical cases presented represent a limited (in sample size) and yet significant qualitative analysis of the *in vivo *efficacy of CS-Platelet.

Newly developed biomaterials intended for clinical use in bone regenerative therapy should be osteoinductive, easy to handle, suitable for an *in situ *application, available on a large scale, safe, and cost effective. CS-Platelet, based on the inexpensive Calcium Sulfate and on the patient's own PRP, meets all of these requirements. In addition, because CS-Platelet is initially fluid and later solidifies, it may be used as an injectable biomaterial for minimally invasive surgeries. Thus, the discovery of CS-Platelet may represent a cost-effective breakthrough in translational medicine and an alternative or an adjuvant to the currently available bone regenerative therapies.

Further and more extended animal and human studies may be needed to effectively translate the findings of the present study into an established and effective protocol for bone regenerative therapy.

## Competing interests

On August 2002, Dr. Giuseppe Intini, Dr. Libuse Bobek, Dr. Sebastiano Andreana and Dr. Rosemary Dziak have submitted a US and International patent application named "Tissue implants and methods for making and using same". The application relates to CS-Platelet and is currently under review by the United States Patent and Trademark Office (USPTO). No patent has been released, yet. Dr. Giuseppe Intini, Dr. Libuse Bobek, Dr. Sebastiano Andreana, and Dr. Rosemary Dziak and a manufacturer and distributor of dental products entered into an agreement pursuant to which the corporation paid certain patent costs in consideration of certain rights in the invention. Dr. Intini and the corporate party to the agreement and others are discussing opportunities for commercialization of the invention described in the Work.

## Authors' contributions

GI wrote the manuscript. GI and SA performed all the animal studies. FEI, GI, and RJB performed the human clinical cases. LAB supervised the experimental work and assisted with the writing of the manuscript. All authors read and approved the final manuscript.
